# Inhibition of cytochrome P450 epoxygenase promotes endothelium-to-mesenchymal transition and exacerbates doxorubicin-induced cardiovascular toxicity

**DOI:** 10.1007/s11033-024-09803-z

**Published:** 2024-07-27

**Authors:** Hevna Dhulkifle, Lubna Therachiyil, Maram H. Hasan, Tahseen S. Sayed, Shahd M. Younis, Hesham M. Korashy, Huseyin C. Yalcin, Zaid H. Maayah

**Affiliations:** 1 Department of Pharmaceutical Sciences, College of Pharmacy, QU Health Sector, Qatar University, 2713 Doha, Qatar; 2 Translational Research Institute, Hamad Medical Corporation, Doha, Qatar; 3 Biomedical Research Center, QU Health Sector, Qatar University, 2713 Doha, Qatar; 4 Department of Biomedical Sciences, College of Health Sciences, QU Health Sector, Qatar University, 2713, Doha, Qatar

**Keywords:** Doxorubicin, Endothelial, Mesenchymal, Cytochrome p450, Epoxyeicosatrienoic acids

## Abstract

**Background:**

Doxorubicin (DOX) is a potent chemotherapy widely used in treating various neoplastic diseases. However, the clinical use of DOX is limited due to its potential toxic effect on the cardiovascular system. Thus, identifying the pathway involved in this toxicity may help minimize chemotherapy risk and improve cancer patients’ quality of life. Recent studies suggest that Endothelial-to-Mesenchymal transition (EndMT) and endothelial toxicity contribute to the pathogenesis of DOX-induced cardiovascular toxicity. However, the molecular mechanism is yet unknown. Given that arachidonic acid and associated cytochrome P450 (CYP) epoxygenase have been involved in endothelial and cardiovascular function, we aimed to examine the effect of suppressing CYP epoxygenases on DOX-induced EndMT and cardiovascular toxicity in vitro and in vivo.

**Methods and Results:**

To test this, human endothelial cells were treated with DOX, with or without CYP epoxygenase inhibitor, MSPPOH. We also investigated the effect of MSPPOH on the cardiovascular system in our zebrafish model of DOX-induced cardiotoxicity. Our results showed that MSPPOH exacerbated DOX-induced EndMT, inflammation, oxidative stress, and apoptosis in our endothelial cells. Furthermore, we also show that MSPPOH increased cardiac edema, lowered vascular blood flow velocity, and worsened the expression of EndMT and cardiac injury markers in our zebrafish model of DOX-induced cardiotoxicity.

**Conclusion:**

Our data indicate that a selective CYP epoxygenase inhibitor, MSPPOH, induces EndMT and endothelial toxicity to contribute to DOX-induced cardiovascular toxicity.

**Supplementary Information:**

The online version contains supplementary material available at 10.1007/s11033-024-09803-z.

## Introduction

Doxorubicin (DOX) is a potent anthracycline chemotherapy broadly used in the treatment of various neoplastic diseases [23] including, but not limited to, breast, lung, gastric, ovarian, thyroid, lymphoma, multiple myeloma, and sarcoma since the 1970s [37]. However, the clinical use of DOX is limited due to its potential toxic effect on the cardiovascular system [30]. Despite extensive research over the last couple of decades [11], the pathogenic mechanisms responsible for DOX-induced cardiovascular toxicity are still ambiguous. Thus, there is an obvious need to explore how DOX damages the cardiovascular system and identify a target that can be modified by drugs and, therefore, reduce chemotherapy-induced cardiovascular damage.

While previous works focused on the direct effect of doxorubicin on cardiac cells [11], recent studies have turned toward endothelium as a novel target for DOX-induced cardiovascular toxicity [23]. Given the systemic administration of DOX, endothelium serves as the primary cellular contact and is thus exposed to elevated DOX concentrations [11]. This exposure to high concentration of DOX promotes a phenotypic change of the endothelium in which endothelial cells lose their features and take on mesenchymal properties in a process known as Endothelium-to-Mesenchymal Transition (EndMT), resulting in endothelial dysfunction, thereby precluding the endothelium from protecting the cardiovascular system [31]. However, the molecular mechanism responsible for DOX-induced EndMT remains unknown.

Notably, one of the small molecules critically involved in the maintenance of endothelial function is epoxyeicosatrienoic acids (EETs) [44]. These endothelium-derived factors, 5,6-, 8,9-, 11,12-, and 14,15-EET are products of olefin epoxidation of the arachidonic acid (AA) by cytochrome P450 (CYP) epoxygenase enzymes [17]. CYP2B, CYP2C, and CYP2J are the main CYPs among the CYP epoxygenase array facilitating the formation of EETs [17]. Importantly, CYP epoxygenase/EETs assume a pivotal role in cardiovascular protection, exerting multifaceted impacts on the endothelium, blood vessels and cardiomyocytes [17]. Specifically, studies have linked CYP epoxygenase/EETs to vasodilation [13] and protection against inflammation [18], EndMT [41], atherosclerosis [40] apoptosis [20] and hypertension [32]. Additionally, endothelial overexpression of CYP epoxygenase, CYP2J2, ameliorates myocardial infarction-induced heart failure [46]. On the other hand, pharmacological inhibition of CYP epoxygenase using a specific inhibitor such as, N-methylsulfonyl-6-(2-propargyloxyphenyl) hexanamide (MSPPOH) or disruption of *CYP2J2* gene exhibits a detrimental effect on the cardiovascular system [3, 33, 45].

Despite the recognized link between CYP epoxygenase and cardiovascular diseases (CVDs) and their role in endothelial function, there’s a lack of studies on the involvement of CYP epoxygenase in DOX-induced endothelial toxicity and EndMT [23]. Given that there is a known connection between CYP epoxygenase and the CV system [25], it is possible that the CYP epoxygenase pathway may play a physiological protective role against DOX-induced endothelial toxicity and EndMT. Thus, we aim to investigate whether suppressing CYP epoxygenase would promote DOX-induced endothelial toxicity and EndMT, thereby exacerbating DOX-induced cardiovascular toxicity.

## Materials and methods

### Materials

Quantitative real-time PCR (qRT-PCR) primers and Gibco Dulbecco’s Modified Eagle Medium F-12 Nutrient Mixture (DMEM/F12) were purchased from Thermo Fisher (Thermo Fisher Scientific, MA, US). EA. hy926, a human endothelial cell line, was purchased from American Type Cell Culture ((ATCC, cat# CRL-2922), Manassas, VA). RIPA Lysis and Extraction Buffer were obtained from Invitrogen (Invitrogen®, Carlsbad, CA, USA).

### Cell culture

EA. hy926 cells were grown in a 5% CO_2_ humidified environment at 37 °C in 75cm^2^ tissue culture flasks with DMEM/F12 (Thermo Fisher Scientific, MA, US). When the cells reached the desired confluence (~ 80%), they were incubated with DOX and/or MSPPOH for 24 h.

### Cell viability

The ability of live cells to convert 3-[4,5-dimethylthiazol-2-yl]-2,5-diphenyl tetrazolium bromide (MTT) to coloured formazan crystals was used to evaluate the effects of DOX and MSPPOH on cell viability, as previously described [27]. The proportion of viable cells was calculated relative to control wells labeled as 100%.

### Cell morphology

EA. hy926 cells were grown in 6-well plates and incubted with 2 μM DOX and/or 50 μM MSPPOH for 24 h to examine their effects on EndMT. Unlike cobblestone monolayers of endothelial cells, EndMT is characterized by elongated spindle-like cells [31]. The cell morphology was quantified by counting the ratio of mesenchymal, i.e., elongated spindle-like cells, to endothelial cells [31]. The concentration of DOX we used in our study is consistent with the previous literature [31] that shows that DOX induced EndMT in endothelial cells. Also, it is comparable to a concentration of 1 µg/ ml (~ 1.72 µM) of DOX that has been reported in the peripheral blood of cancer patients [34].

### Flow cytometric analysis

Apoptosis was examined using Dual Staining of Annexin V and Propidium iodide (PI) (BD Biosciences, San Jose, CA, USA) according to the manufacturer’s procedure as described previously [35]. Briefly, we incubated EA. hy926 cells with 2 μM DOX and/or 50 μM MSPPOH for 24 h. Then, we washed them with phosphate buffer saline (PBS) and reconstituted them in the dark in FOTC-labeled annexin V and PI-containing binding solution for 15 min. The collected cells were then examined consequently with a flow cytometer.

### Whole protein extraction

We incubated EA. hy926 cells with 2 μM DOX and/or 50 μM MSPPOH for 24 h. Thereafter, we washed the cells with PBS, and we added RIPA buffer supplemented with a Halt protease-phosphatase inhibitor cocktail (1X) to extract the protein. Then, we scraped the cells for 30 min on ice, transferred the extract into a fresh Eppendorf tube, centrifuged the tubes at 14,000×*g*, 4 °C for 15 min, and transferred the supernatants into a new tube. Finally, we used a Rapid Gold BCA assay kit (Thermo Scientific, MA, USA) to quantify the protein as described previously [36].

### Liquid chromatography–tandem mass spectrometry (LC–MS/MS) analysis

We prepared the samples for mass spectrometry analysis using a previously described method [36]. Using a TimsTOF Pro mass spectrometer (Bruker Daltonics, Germany) coupled to a nano-liquid chromatography system nano-elute (Bruker Daltonics, Germany), we carried out trip ion mobility mass spectrometry, which includes MS and MS/MS, as previously described [36].

### MS and MS/MS data processing and analysis

The built-in search engine Andromeda [9] in the MaxQuant software (version 2.1.4.0) was used to process the MS/MS raw data according to standard workflow [38]. We used UniProtKB/Swiss-Prot human database to identify proteins with fixed and variable modifications as described previously [36]. Also, we used the MaxLFQ label-free quantitation method to extract maximum quantification information in MaxQuant as described previously [10]. Normalized spectral intensity (LFQ intensity) was then used to calculate protein abundance.

We used the Perseus software to carry out the data analysis [39]. After being imported from the MaxQuant analysis, the LFQ intensities were converted to log2(x). Every protein expressed in each of the three biological replicates underwent at least one conditional comparison in the statistical analysis. For each missing LFQ intensity value, values from the normal distribution (width = 0.3, downshift = 1.8) were substituted. Finally, we used a two-tailed student’s t test for protein quantification, where we calculated the statistically significant abundance using an adjusted *p* value < 0.05. We used Reactome pathway analysis to analyze the functional enrichment of the differentially expressed proteins (DEPs) as described previously [36].

### Zebrafish husbandry

This experiment was carried out using wild-type zebrafish embryos (AB strain). All animal studies were conducted in accordance with international guidelines and the polices required by Qatar University and the Department of Research in the Ministry of Public Health for the use of zebrafish in experimental studies under the approval of the Institutional Animal Care and Use Committee (IACUC) (QU-IACUC 020/2020-REN2).

Zebrafish embryos were housed in a 10-mm petri plate at 28.5 °C. We used a Zeiss SteREO Discovery V8 microscope and a Hamamatsu Orca Flash High-speed camera to monitor the survival and the morphology changes at 24-h intervals over 3 days. Hatching, survival, and any changes have been monitored and documented. Dead embryos were removed immediately after they were discovered.

### Measurement of cardiovascular function and structure

We treated the zebrafish embryos at 24 h post-fertilization (24 hpf) as most given drugs are able to pass via the chorion and thus accumulate inside the embryos [16]. We followed a previously established protocol to induce cardiotoxicity in zebrafish using DOX [21]. Briefly, at 24 hpf, the fish embryos were arbitrarily divided into four groups that were incubated with either vehicle (n = 10), 100 μM of DOX (n = 10), 50 μM of MSPPOH (n = 10), or a combination of 100 μM of DOX and 50 μM of MSPPOH (n = 10) for 72 h. We kept the embryos in the dark at 28.5 °C.

The treated fish’s body structure, cardiovascular function, and survival rate were evaluated at 72 hpf. In brief, ten fish larvae at random from each group were examined under a microscope after stabilizing them with 3% methylcellulose. Each fish had a 10-s bright field film of its heart and body taken at 100 frames per second (fps). The flow velocity, arterial pulse, and vessel diameter were measured in the exact location of the dorsal aorta (DA) and the posterior cardinal vein (PCV) using the Viewpoints MicroZebralab version 3.6 software [43]. Vascular blood flow velocity measurements are commonly used to evaluate cardiovascular function in zebrafish [43]. A slower blood flow velocity and/or lower cardiac output suggest deterioration in cardiovascular function [43]. The cardiovascular function parameters were calculated using formulas illustrated in Supplementary Table 1.

### Quantitative real-time PCR (qRT-PCR)

We isolated total RNA from EA. hy926 or Zebrafish larvae by TRIzol reagent (Invitrogen®, Carlsbad, CA, USA) as described previously [24, 26]. The Nano spectrophotometer was used to assess total RNA concentration and purity. Complementary DNA (cDNA) was generated using the High-Capacity cDNA Reverse Transcription Kit (Applied Biosystems, Foster City, CA, USA), according to the manufacturer’s instructions. We used QuantStudio 5 to quantify the expression of mRNA as shown previously [12]. Supplementary Table 2 listed the primer sequences that have been used in this study. The relative gene expression ΔΔCt method was used to analyze qRT-PCR data. We calculated the fold change between the mRNA levels using the previously described equation formula: fold change = 2^−Δ(ΔCt)^, where ΔCt = Ct_target_ − Ct_β-actin_ and Δ(ΔCt) = ΔCt_treated_ − ΔCt_untreated_ as described previously [12]. Our reference primers, β-Actin, and rpl13a, for human and zebrafish, respectively, were used to correct the fold change between the mRNA expressions. Lastly, we used 2^−△CT^ formula to calculate the basal expression level of CYP isoforms in the endothelial cells.

### Statistical analysis

For our statistical study, we used GraphPad Prism (version 7.04) from GraphPad Software, Inc. in La Jolla, California. Results are displayed as mean ± SEM. Tukey Kramer’s post-hoc multiple comparison test was employed following one-way analysis of variance (ANOVA) to evaluate the statistical differences between the groups. A probability value found that was considered significant was less than 0.05.

## Results

### Doxorubicin promotes endothelial-to-mesenchymal transition in human endothelial cells

**Fig. 1:**
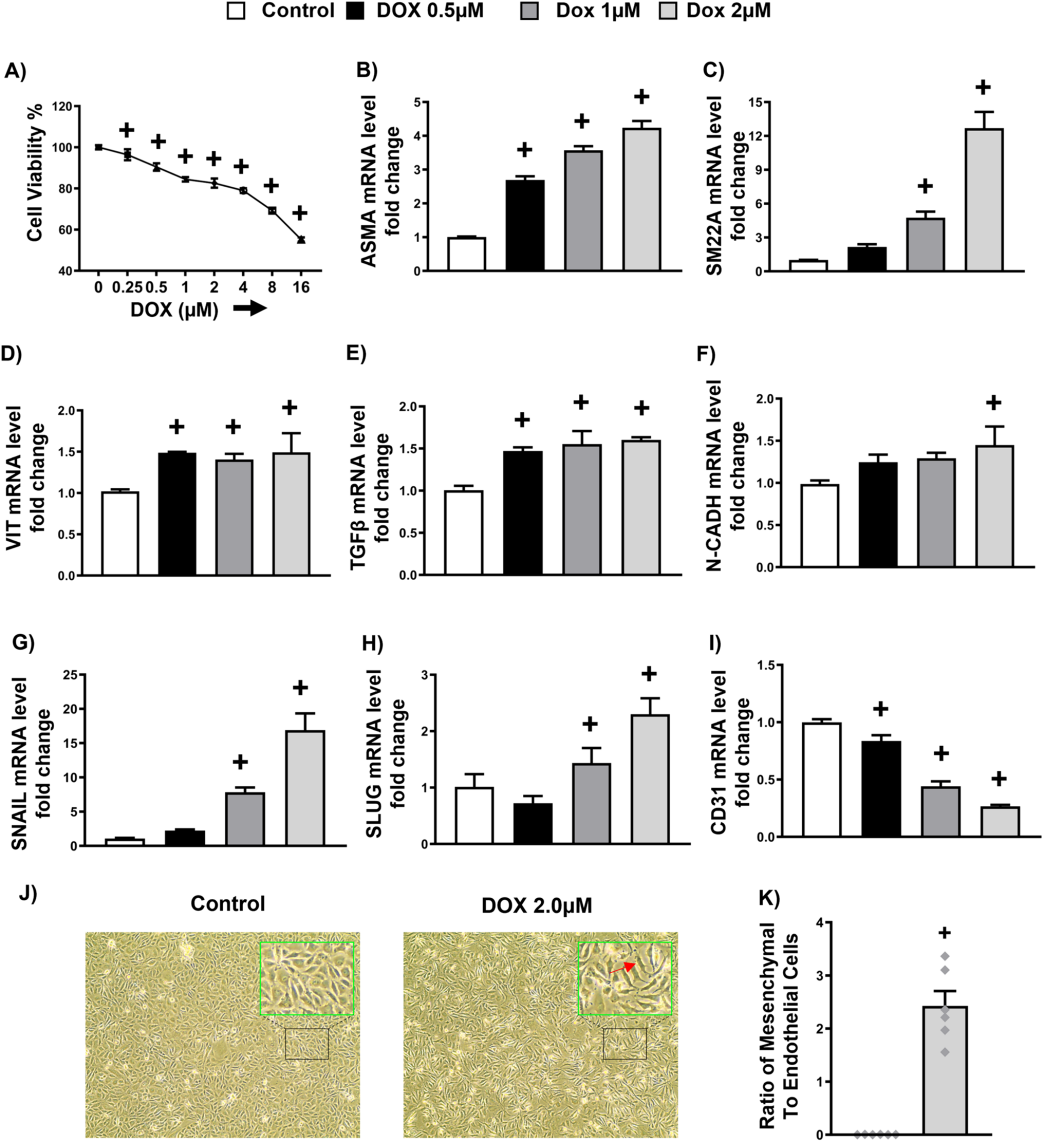
Doxorubicin promotes Endothelial-to-mesenchymal transition in human endothelial cells. **A** MTT Assay. **B**–**I** Quantification of mRNA expression levels by qRT-PCR. **B** Alpha-Smooth Muscle Actin (ASMA), **C** Smooth muscle protein 22 alpha (SM22A), **D** VIMENTIN (VIT), **E** Transforming growth factor-β (TGFβ), **F** N-CADHERIN (N-CADH), EndMT associated transcription factors (**G**) SNAIL (**H**) SLUG and (**I**) CD31. **J** Representative images of cells treated with either vehicle or 2 µM DOX. **K** The ratio of Mesenchymal cells to Endothelial cells. Results are shown as means ± SEM. An unpaired t-test was used to determine if the ratio of mesenchymal-to-endothelial cells in groups treated with 2 µM DOX was significantly different from the control. A one-way analysis of variance (ANOVA), followed by Tukey Kramer’s post hoc multiple comparison test, was used to assess if different concentrations of DOX displayed a significant difference from those of the control group. ^+^*p* < 0.05 vs control

We tested whether DOX could develop the transition of endothelial cells into mesenchymal cells, i.e., EndMT in our endothelial cells, EA hy926 cells. To do this, we incubated EA hy926 cells with different concentrations of DOX for 24 h. Following the MTT assay, we used 0.5 µM, 1 µM and 2 µM concentrations of DOX for our experiment in the human endothelial cells as more than 80% of the cells are viable, and these concentrations are within the plasma levels in humans [34]. Using these concentrations, we then examined the expression levels of endothelial and mesenchymal markers. Notably, we found that DOX significantly upregulated the expression of EndMT markers, including the levels of smooth muscle actin, ASMA and SM22A (Fig. 1B, C), VIMENTIN  (Fig. 1D), transforming growth factor-β (TGFβ) (Fig. 1I), N-CADHERIN (CDH2) (Fig. 1F), SNAIL (SNAI1) (Fig. 1G), and SLUG (SNAI2) (Fig. 1H) in a concentration-dependent manner when compared to control. In contrast, DOX significantly downregulated the expression of endothelial cell marker CD31 in all tested concentrations when compared to the control. Given that the 2 µM concentration of DOX demonstrated the maximum upregulation of EndMT markers, we use this concentration to examine the morphological changes of the endothelial cells. Consistent with previous studies [14, 29], we found that DOX-induced EndMT resulted in increased cellular gap and altered the arrangement and shape of the cells from a monolayer cobblestone to disorganized and long spindle-like cells (Fig. 1J) [29, 42]. Overall, our findings indicated that DOX promoted EndMT.

### Doxorubicin-induced endothelial-to-mesenchymal transition is associated with the upregulation of inflammation and apoptosis markers

**Fig. 2:**
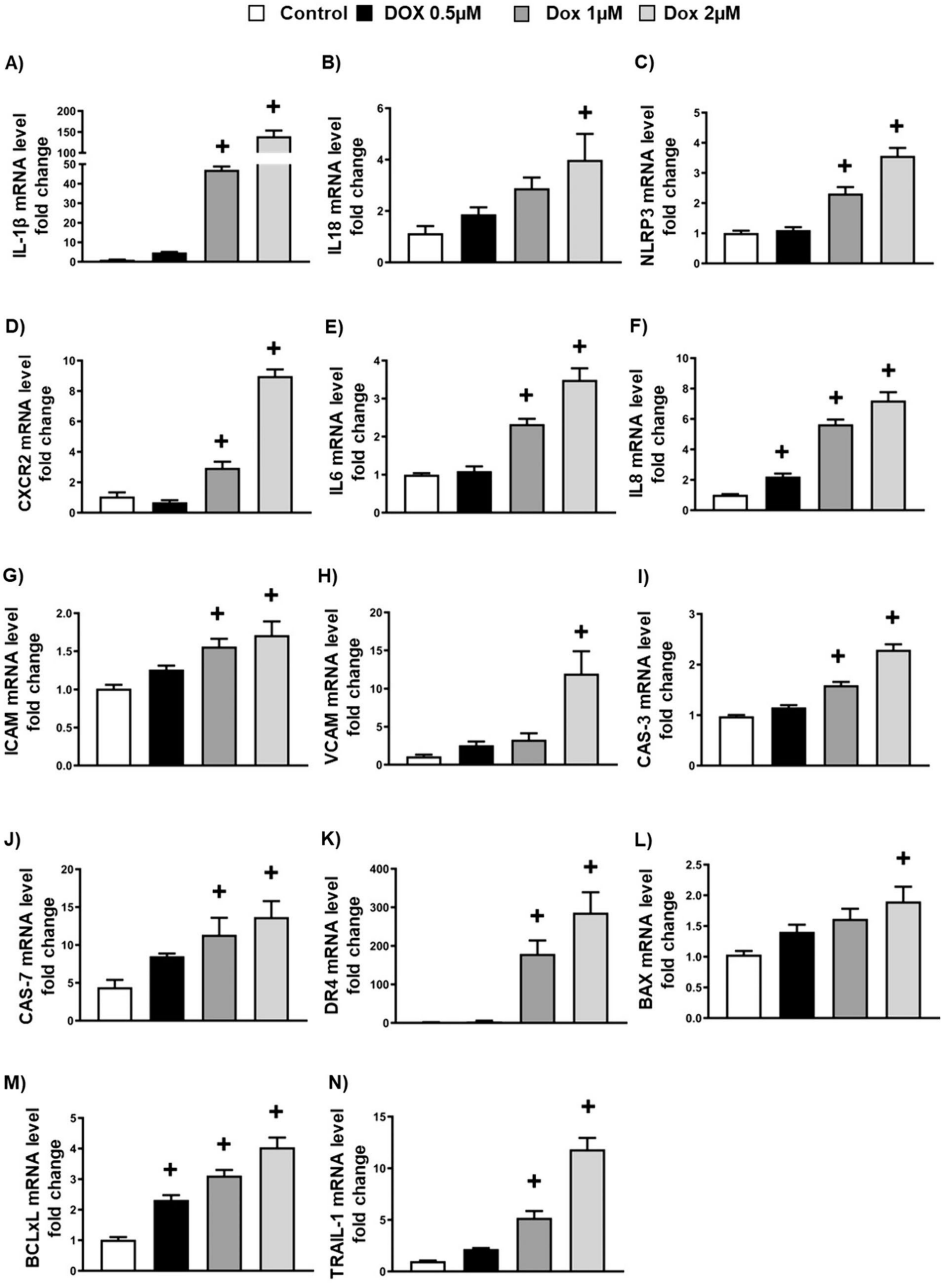
Doxorubicin-induced Endothelial-to-Mesenchymal Transition is associated with the upregulation of inflammation and apoptosis markers. **A**–**N** Quantification of mRNA expression levels by qRT-PCR. **A** IL-1β, **B** IL-18, **C** NLRP3, **D** CXCR2, **E** IL6, **F** IL8, **G** ICAM, **H** VCAM, **I** CASPASE 3 (CAS-3), **J** CASPASE 7 (CAS-7), **K** Death receptor 4 (DR4), **L** BAX, **M** BCL-xL, **N** TRAIL-1. Results are shown as means ± SEM. A one-way analysis of variance (ANOVA), followed by Tukey Kramer’s post hoc multiple comparison test, was used to assess if different concentrations of DOX displayed a significant difference from those of the control group. ^+^*p* < 0.05 vs control

Since EndMT plays a pivotal role in the development of inflammation and apoptosis of endothelial cells [31], we investigated whether DOX-induced EndMT is accompanied by an increase in inflammation and apoptosis. To test this, we incubated the endothelial cells with different concentrations of DOX for 24, and then we examined the mRNA gene expression levels of inflammation and apoptotic markers. Our results show that treatment with DOX significantly increased the mRNA expression of IL-1β, IL-18, NLRP3, CXCR2, IL-6, IL-8, ICAM, and VCAM in a concentration-dependent manner compared to control (Fig. 2A–H). Furthermore, apoptotic markers, including CASPASE 3 and 7, death receptor 4 (DR4), BAX, BCL-xL, and TRAIL-1, were significantly upregulated in a concentration-dependent manner in cells treated with DOX (Fig. 2I–N). Collectively, these results indicate that DOX-induced EndMT is associated with the upregulation of inflammation and apoptotic markers.

### Doxorubicin-induced endothelial-to-mesenchymal transition is exacerbated by CYP epoxygenase inhibition

**Fig. 3:**
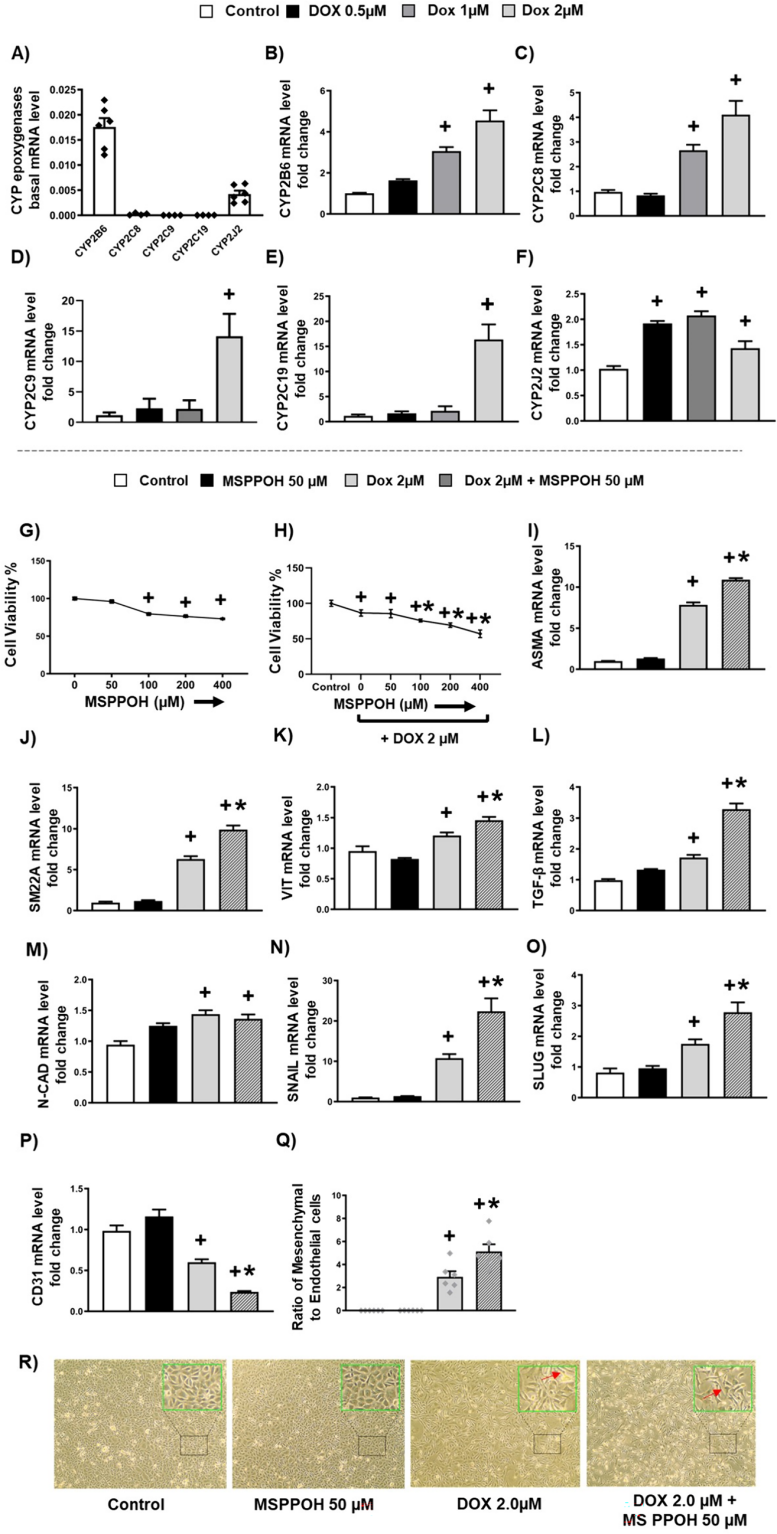
Doxorubicin-induced Endothelial-to-Mesenchymal Transition is exacerbated by CYP epoxygenase inhibition. **A** Basal levels of CYP epoxygenase mRNA. **B**–**F** Quantification of mRNA expression levels by qRT-PCR. **B** CYP2B6, **C** CYP2C8, **D** CYP2C9, **E** CYP2C19 and **F** CYP2J2. **G** MTT Assay for MSPPOH **H** MTT Assay for MSPPOH in combination with DOX. **I**–**P** Quantification of mRNA expression levels by qRT-PCR. **I** Alpha-Smooth Muscle Actin (ASMA), **J** Smooth muscle protein 22 alpha (SM22A), **K** VIMENTIN (VIT), **L** Transforming growth factor-β (TGFβ), **M** N-CADHERIN (N-CAD), EndMT associated transcription factors (**N**) SNAIL (**O**) SLUG and (**P**) CD31. **R** Representative images of cells. **K** The ratio of Mesenchymal cells to Endothelial cells. Results are shown as means ± SEM. We used one-way analysis of variance (ANOVA) followed by Tukey Kramer’s post hoc multiple comparison tests.  ^+^*p* < 0.05 vs control; *p < 0.05 vs DOX

CYP epoxygenase is known to play a critical role in the maintenance of endothelial cells’ health and homeostasis [44]. Therefore, we assumed that CYP epoxygenase is constitutively expressed in endothelial cells, EA hy926 cells. Consistent with this, our results show that while CYP epoxygenase, including CYP2B6, CYP 2J2, and CYP2C, were all expressed in EA hy926 cells, CYP2B6 followed by CYP2J2 demonstrated a relatively higher expression level (Fig. 3A). Notably, the expressions of CYP2B6, CYP2C8, CYP2C9, CYP2C19 and CYP 2J2 were all upregulated in EA hy926 cells by DOX in a concentration-dependent manner (Fig. 3B, C, D, E, F). Since the most significant changes in CYP expression were observed at 2 µM DOX, this concentration was selected to perform all subsequent experiments.

Given that CYP epoxygenase is expressed in endothelial cells and upregulated by DOX treatment, we hypothesized that CYP epoxygenase plays a physiologic defensive impact on DOX-induced endothelial toxicity like EndMT. If this is the case, we would predict that a selective CYP epoxygenase inhibitor, MSPPOH, might reduce this physiologic protective effect of CYP epoxygenase and exacerbate DOX-induced endothelial toxicity like EndMT. To test this hypothesis, we conducted in vitro experiments to test the impact of MSPPOH on EndMT in endothelial cells incubated with 2 µM DOX for 24 h. Following the MTT Assay, we selected a 50 µM concentration of MSPPOH for the EA hy926 experiment as it did not reduce endothelial cell viability when it is either used alone or in combination with DOX (Fig. 3G, H). Notably, we found that DOX-treated cells demonstrated a significant upregulation of EndMT markers, including ASMA, SM22A, VIMENTIN, TGF-β, N-CADHERIN, SNAIL, and SLUG (Fig. 3I–O) and a substantial downregulation of endothelial cell marker, CD31, compared to control (Fig. 3P). Consistent with our hypothesis, our results show that MSPPOH, a selective CYP epoxygenase inhibitor, significantly elevated the expression of EndMT markers, including ASMA, SM22A, VIMENTIN, TGF-β, N-CADHERIN, SNAIL, and SLUG (Fig. 3I–O) and significantly reduced the expression of endothelial cell marker, CD31 (Fig. 3P), in endothelial cells incubated with DOX. Since DOX-induced EndMT cells are also associated with morphological changes, we also examined whether MSPPOH exacerbated the morphological modifications of DOX-treated endothelial cells. Interestingly, MSPPOH significantly disassembled the cobblestone characteristic of endothelial cells into irregular and elongated mesenchymal cells and further elevated the ratio of mesenchymal cells to endothelial cells of DOX-treated endothelial cells (Fig. 3Q, R). Collectively, our data indicates that inhibition of CYP epoxygenase exacerbates DOX-induced EndMT.

### Doxorubicin-induced inflammation and apoptosis are exacerbated by CYP epoxygenase inhibition

**Fig. 4:**
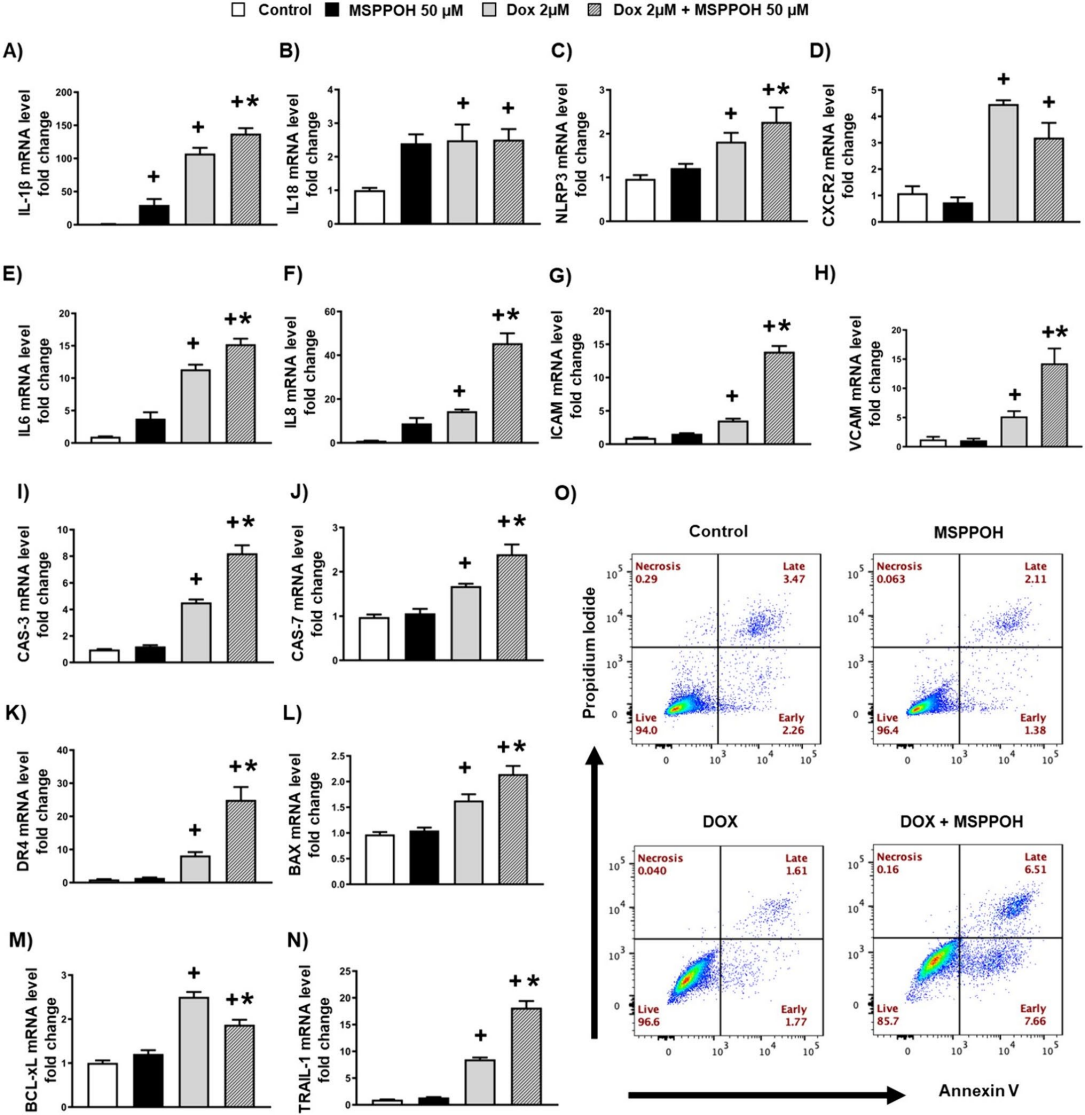
Doxorubicin-induced inflammation and apoptosis are exacerbated by CYP epoxygenase inhibition. **A**–**N** Quantification of mRNA expression levels by qRT-PCR. **A** IL-1β, **B** IL-18, **C** NLRP3, **D** CXCR2, **E** IL6, **F** IL8, **G** ICAM, **H** VCAM, **I** CASPASE 3 (CAS-3), **J** CASPASE 7 (CAS-7), **K** Death receptor 4 (DR4), **L** BAX, **M** BCL-xL, **N** TRAIL-1. **O** Annexin V/propidium iodide (PI) staining and flow cytometry were used to determine apoptosis in the various cells. Results are shown as means ± SEM. We used one-way analysis of variance (ANOVA) followed by Tukey Kramer’s post hoc multiple comparison test.  ^+^*p* < 0.05 vs control; *p < 0.05 vs DOX

Since DOX-induced EndMT was associated with the upregulation of inflammation and apoptotic markers, we tested whether MSPPOH could also exacerbate DOX-induced inflammation and apoptosis in human endothelial cells. Notably, we found that MSPPOH further upregulated the levels of inflammatory markers like IL1β, IL-18, NLRP3, CXCR2, IL-6, IL-8, ICAM, and VCAM (Fig. 4A–H) in endothelial cells incubated with DOX suggesting that MSPPOH exacerbates DOX-induced inflammation. Similar to this, MSPPOH also elevated proapoptotic markers, including CASPASE 3 and 7, DR4, BAX, and TRAIL-1 (Fig. 4I, J, K, L, N) and reduced antiapoptotic marker, BCl-xL (Fig. 4M), in endothelial cells incubated with DOX. Consistent with the apoptotic markers, our flow cytometry data confirmed that MSSPOH increased the number of apoptotic cells in our endothelial cells incubated with DOX. Overall, these results suggest that MSPPOH exacerbated DOX-induced inflammation and apoptosis (Fig. 4O).

### CYP epoxygenase inhibition alters the proteomic profile of doxorubicin-treated endothelial cells

**Fig. 5:**
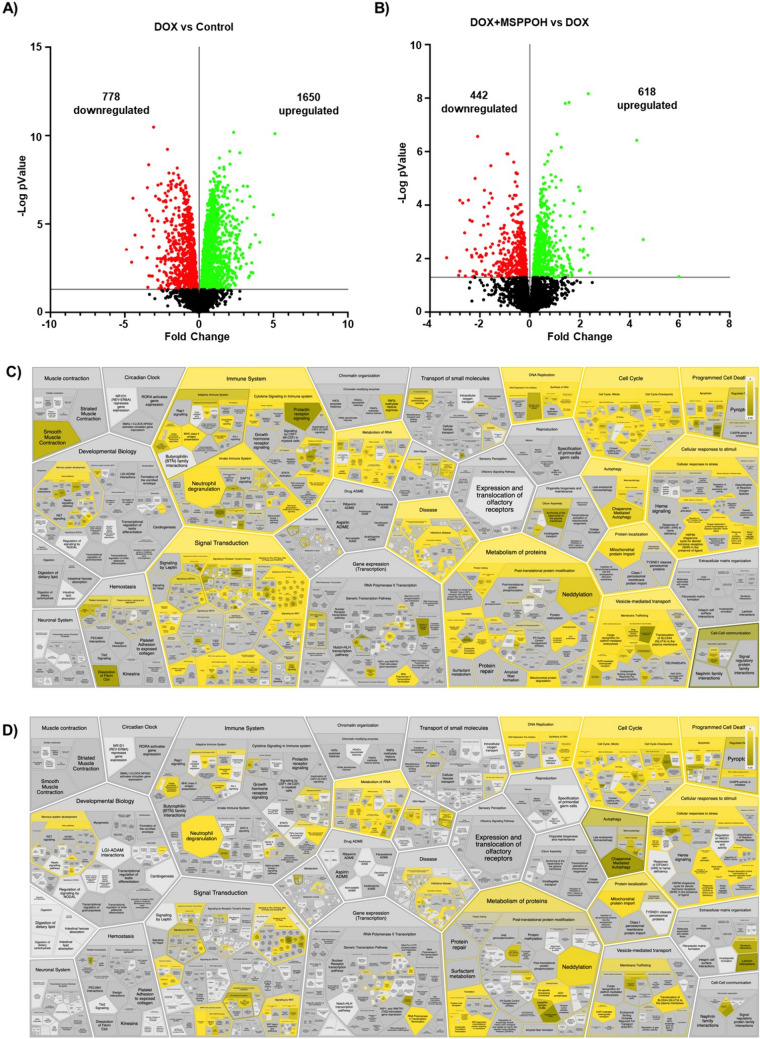
Effect of CYP Epoxygenase Inhibition on the Proteomic Profile of Doxorubicin-Treated Endothelial Cells. Volcano plots of differentially expressed proteins (DEPs) were obtained. The x-axis represents the log2 expression fold-change in **A** DOX vs Control and **B** DOX + MSPPOH vs DOX. Up-regulated genes are shown in green, while down-regulated genes are in red. Voronoi diagram depicting reaction analysis and Reactome pathways associated with the differentially expressed proteins in **C** DOX vs Control and **D** DOX + MSPPOH vs DOX. The gradient of colours depicts significance, with the most significance highlighted in bright yellow (P = 0.00) and the least in dark yellow (P = 0.05)

To investigate the effect of CYP epoxygenase inhibitor, MSPPOH, on DOX-induced endotheliotoxicity at the proteomic level, we treated EA hy926 cells with DOX with and without MSPPOH for 24 h. We then performed LC–MS/MS and analyzed the results to find the differentially expressed proteins (DEP) using MaxQuant analysis. Notably, our findings indicate that 2428 proteins were significantly changed in endothelial cells treated with DOX compared to control (Fig. 5A). Of these, 778 proteins were found to be upregulated while 1650 proteins were downregulated in endothelial cells treated with DOX compared to control (Fig. 5A). We also observed that the treatment of cells with a combination of DOX and MSPPOH led to the upregulation of 618 proteins and the downregulation of 442 proteins compared to DOX treatment alone. Upon analyzing the Reactome Pathway Database, we found a significant alteration in proteins related to RNA and protein metabolism, cell cycle, programmed cell death, cellular response to stimuli, and immune system as a result of DOX treatment compared to the control group (Fig. 5C). Of interest, proteins involved in the cell cycle, programmed cell death, cellular response to stimuli and immune system pathways were further modulated in endothelial cells incubated with a combination of DOX and MSPPOH compared to the cells treated with DOX alone (Fig. 5D).

We further analyzed our proteomic profile for proteins involved in EndMT, inflammation and apoptosis. Notably, in a manner similar to what we have observed at the mRNA level, DOX-treated cells have shown a significant increase in the expression of specific EndMT-related protein expression and a decrease in some endothelial cell markers (Table 1). Interestingly, when endothelial cells were treated with a combination of DOX and MSPPOH, the proteins involved in EndMT, including Caldesmon, Calumenin, Caveolin-1, Caveolin, Cadherin-2 (N-cadherin), Cyclin-dependent kinase 2, Collagen alpha-1(V) chain, Endothelin-converting enzyme 1, Endothelial differentiation-related factor 1, Latent-transforming growth factor beta-binding protein 4, Matrix-remodeling-associated protein 7, Paxillin, Platelet endothelial aggregation receptor 1, Serpin H1, Transforming growth factor beta-1-induced transcript 1 protein, Cdc42-interacting protein 4, Ubiquitin-conjugating enzyme E2 C, Transcriptional coactivator YAP1, and Zyxin were significantly upregulated compared to the cells treated with DOX alone (Table 1). On the other hand, endothelial cell markers like Alpha-adducin, Ephrin-B1, Protocadherin alpha-3, and Ubiquitin carboxyl-terminal hydrolase were significantly downregulated in the cells incubated with a combination of DOX and MSPPOH compared to the cells treated with DOX alone (Table 1). Unexpectedly, while DOX did not significantly affect Platelet endothelial cell adhesion molecule (i.e., CD31) and significantly reduced the protein expression of vimentin compared to control (Table 1), cells treated with a combination of DOX and MSPPOH demonstrated a decrease in the level of vimentin and an increase in the level of CD31 (Table 1). Nevertheless, our overall proteomic data suggests that inhibition of CYP epoxygenase exacerbates DOX-induced EndMT at the protein level.

**Table 1:** Proteomic Profile for endothelial cells treated with DOX and DOX + MSPPOH

Gene name	Protein IDs	Protein names	DOX vs control		Dox + MSPPOH vs DOX	
			Fold change	p value	Fold change	p value
Endothelial-to-mesenchymal transition						
ADD1	E7EV99	Alpha-adducin	0.812	2.08E−07	− 0.276	0.00126
CALD1	Q05682-5	Caldesmon	1.2	2.19E−06	0.543	0.00113
CALU	O43852	Calumenin	0.551	9.68E−05	0.205	0.0387
CAV1	Q03135	Caveolin-1;Caveolin	0.423	0.000406	0.368	0.00108
CDH2	C9J126	Cadherin-2 (N-cadherin)	1.05	2.13E−06	0.226	0.0531
CDK2	P24941	Cyclin-dependent kinase 2	0.459	0.0019	0.247	0.0458
COL5A1	P20908-2	Collagen alpha-1(V) chain	1.43	0.000381	1.74	8.71E−05
ECE1	A0A3B3ISF9	Endothelin-converting enzyme 1	1.05	6.71E−07	0.186	0.0662
EDF1	O60869	Endothelial differentiation-related factor 1	1.59	2.72E−07	0.66	0.000386
EFNB1	P98172	Ephrin-B1	0.0135	0.965	− 1.69	0.000266
HYAL2	Q12891	Hyaluronidase-2	3.42	2.78E−07	− 0.567	0.0593
LTBP4	A0A0C4DH07	Latent-transforming growth factor beta-binding protein 4	0.462	0.0103	0.29	0.0742
MXRA7	P84157-2	Matrix-remodeling-associated protein 7	0.354	0.078	0.723	0.0027
PCDHA3	Q9Y5H8-2	Protocadherin alpha-3	0.0241	0.979	− 2.13	0.0372
PEAR1	Q5VY43	Platelet endothelial aggregation receptor 1	0.482	0.0769	0.663	0.0224
PECAM1	P16284-3	Platelet endothelial cell adhesion molecule	1.71	2.81E−07	0.183	0.198
PXN	A0A1B0GTU4	Paxillin	0.465	9.21E−05	0.251	0.00623
SERPINH1	P50454	Serpin H1	0.0595	0.397	0.17	0.0312
TGFB1I1	O43294	Transforming growth factor beta-1-induced transcript 1 protein	2.05	0.000489	0.815	0.0668
TRIP10	Q15642-2	Cdc42-interacting protein 4	0.662	6.43E−06	0.266	0.0052
UBE2C	O00762	Ubiquitin-conjugating enzyme E2 C	3.69	1.08E−06	0.757	0.0477
USP3	Q6JHV3	Ubiquitin carboxyl-terminal hydrolase; Ubiquitin carboxyl-terminal hydrolase 3	0.222	0.566	− 0.976	0.0271
VIM	P08670	Vimentin	− 0.921	6.92E−08	− 0.55	6.45E−06
YAP1	P46937-5	Transcriptional coactivator YAP1	− 0.986	0.0564	1.03	0.048
ZYX	Q15942	Zyxin	0.32	0.00366	0.442	0.000433
Inflammation						
ALCAM	Q13740-2	Activated leukocyte cell adhesion molecule (ALCAM)), CD166 antigen	0.501	0.00101	0.619	0.000221
ANO6	Q4KMQ2	Anoctamin-6	0.614	0.00175	0.72	0.000592
BCAP29	C9IYK6	B-cell receptor-associated protein 29	1.28	0.000694	1.05	0.00257
CD46	P15529-15	Membrane cofactor protein (MCP; CD46)	0.681	0.0334	1.35	0.000705
DPP8	J3KPT0	Dipeptidyl peptidase 8	− 0.171	0.684	− 2.69	8.37E−05
FAS	Q59FU8	Tumor necrosis factor receptor superfamily member 6	0.768	0.0123	0.972	0.00328
FLOT1	O75955	Flotillin-1	0.469	0.000443	0.349	0.00317
FSTL1	Q12841	Follistatin-related protein 1	− 0.0333	0.673	0.859	9.64E−07
ICAM2	J3QRT5	Intercellular adhesion molecule (ICAM) 2	− 0.657	2.45E−06	0.152	0.0434
IFI16	Q16666-3	Gamma-interferon-inducible protein 16	− 0.0187	0.822	0.188	0.0434
IGBP1	P78318	Immunoglobulin-binding protein 1	0.485	0.0248	0.545	0.0143
IL18	Q14116	Interleukin-18	0.935	0.000787	0.286	0.169
ILF2	Q12905	Interleukin enhancer-binding factor 2	− 1.5	5.78E−08	0.301	0.0124
ILK	A0A0A0MTH3	Integrin-linked protein kinase	0.264	0.0545	0.388	0.00986
IRF2BP1	Q8IU81	Interferon regulatory factor 2-binding protein 1	0.589	0.214	− 2.4	0.000366
LRRFIP1	Q32MZ4-3	Leucine-rich repeat flightless-interacting protein 1	0.785	3.18E−05	0.406	0.00361
MCAM	P43121	Cell surface glycoprotein MUC18 (CD146)	0.315	0.0255	1.03	8.83E−06
PHB	P35232	Prohibitin	− 0.103	0.223	− 0.285	0.00529
PHB2	Q99623	Prohibitin-2	− 0.15	0.15	− 0.282	0.0154
TNFRSF10A	F8U8C0	Tumor necrosis factor receptor superfamily member 10A	− 2.36	0.00512	1.72	0.0253
TXLNA (IL14)	P40222	Alpha-taxilin (interleukin-14 (IL-14))	− 0.905	3.13E−06	0.606	8.66E−05
Apoptosis						
ACIN1	S4R3H4	Apoptotic chromatin condensation inducer in the nucleus	0.92	0.0934	1.15	0.0435
ATR	Q13535-2	Serine/threonine-protein kinase ATR	1.49	0.0554	− 1.79	0.0272
AVEN	Q9NQS1	Cell death regulator Aven	0.264	0.581	1.27	0.0218
BCL10	O95999	B-cell lymphoma/leukemia 10	0.736	0.0998	1.06	0.0265
BCLAF1	Q9NYF8-2	Bcl-2-associated transcription factor 1	1.73	2.45E−06	0.548	0.0104
CASP3	P42574	Caspase-3;Caspase-3 subunit p17;Caspase-3 subunit p12	− 0.0909	0.26	0.0458	0.56
CASP7	P55210	Caspase-7;Caspase-7 subunit p20;Caspase-7 subunit p11	0.653	0.000382	0.307	0.0311
CIAPIN1	Q6FI81	Anamorsin	0.345	0.00507	− 0.224	0.0414
DIDO1	Q9BTC0	Death-inducer obliterator 1	0.46	0.0234	0.435	0.0298
GOT1	P17174	Aspartate aminotransferase, cytoplasmic	0.457	0.000117	0.386	0.000408
GOT2	P00505	Aspartate aminotransferase, mitochondrial	0.587	0.000342	0.891	1.27E−05
PDCD5	O14737	Programmed cell death protein 5	0.762	7.04E−05	0.234	0.0674
PYCARD	Q9ULZ3-3	Apoptosis-associated speck-like protein containing a CARD	0.314	0.44	1.25	0.00991
URM1	Q9BTM9-3	Ubiquitin-related modifier 1x	− 0.138	0.735	1.11	0.0202
Oxidative stress						
CAT	P04040	Catalase	0.769	0.000307	0.809	0.00021
GSTO1	P78417	Glutathione S-transferase omega-1	− 0.109	0.21	0.26	0.0101
GSTP1	P09211	Glutathione S-transferase P	− 0.971	0.000519	− 0.667	0.00586
HAGH	Q16775-2	Hydroxyacylglutathione hydrolase, mitochondrial	0.123	0.366	0.471	0.00508
MT2A	P02795	Metallothionein-2;Metallothionein-1G	1.24	7.33E−06	0.406	0.0169
PON2	A0A0J9YXF2	Serum paraoxonase/arylesterase 2	0.102	0.13	0.33	0.000383
PRDX3	P30048-2	Thioredoxin-dependent peroxide reductase, mitochondrial	− 0.196	0.0231	0.281	0.00342
PRDX5	P30044-2	Peroxiredoxin-5, mitochondrial	0.271	0.0158	− 0.281	0.0132
PRDX6	P30041	Peroxiredoxin-6	0.032	0.557	− 0.134	0.0303
SOD1	P00441	Superoxide dismutase [Cu–Zn]	0.639	0.000607	0.28	0.053
TXNL1	O43396	Thioredoxin-like protein 1	0.404	0.00109	0.407	0.00103
TXNRD1	Q16881-4	Thioredoxin reductase 1, cytoplasmic	0.00881	0.891	0.152	0.0375
TXNRD2	A0A182DWF2	Thioredoxin reductase 2, mitochondrial	0.73	0.000102	0.278	0.0357
UBQLN1	Q9UMX0	Ubiquilin-1	0.233	0.0598	0.503	0.00114

In addition to EndMT markers, we found that DOX significantly altered the expression of proteins related to inflammation as compared to control (Table 1). Notably, a combination of MSPPOH and DOX led to a significant upregulation of proinflammatory proteins such as Activated leukocyte cell adhesion molecule (ALCAM, CD166 antigen), Anoctamin-6, B-cell receptor-associated protein 29, CD46, Tumor necrosis factor receptor superfamily member 6, Flotillin-1, Follistatin-related protein 1, Intercellular adhesion molecule (ICAM) 2, Gamma-interferon-inducible protein 16, Immunoglobulin-binding protein 1, Interleukin enhancer-binding factor 2, Integrin-linked protein kinase (ILK), Leucine-rich repeat flightless-interacting protein 1, CD146, Tumor necrosis factor receptor superfamily member 10A, and IL-14 when compared to DOX alone (Table 1). On the other hand, treatment of cells with MSPPOH and DOX significantly downregulated anti-inflammatory proteins like Dipeptidyl peptidase 8, Interferon regulatory factor 2-binding protein 1, Prohibitin, and Prohibitin-2 as compared to cells treated with DOX alone (Table 1). Together, our findings suggest that MSPPOH further aggravated DOX-induced inflammation at the protein level.

Consistent with our mRNA and flow cytometry data, we found that MSPPOH worsened DOX-induced apoptosis in our endothelial cells by increasing the protein expression of various apoptosis-related proteins such as Apoptotic chromatin condensation inducer in the nucleus (ACIN1), Cell death regulator Aven, B-cell lymphoma/leukemia 10, Bcl-2-associated transcription factor 1, Caspase-7, Death-inducer obliterator 1, Aspartate aminotransferase, a Programmed cell death protein 5, Apoptosis-associated speck-like protein containing a CARD, and Ubiquitin-related modifier 1× when compared to DOX alone. Also, MSPPOH significantly decreased the expression of antiapoptotic proteins like Serine/threonine-protein kinase ATR and Anamorsin in cells treated with DOX compared to DOX alone (Table 1).

Since DOX is known to cause oxidative stress in numerous experimental models, we also investigated if the detrimental effect of MSPPOH in our endothelial cell model is also linked with further aggravation of oxidative stress. Using our proteomics profile, we found that MSPPOH significantly altered the levels of various oxidative stress markers such as Catalase, Glutathione S-transferase omega-1, Glutathione S-transferase P, Hydroxyacylglutathione hydrolase, mitochondrial Metallothionein-2, Serum paraoxonase, Thioredoxin-dependent peroxide reductase, Peroxiredoxin-5, Peroxiredoxin-6, Superoxide dismutase [Cu–Zn], Thioredoxin-like protein 1, Thioredoxin reductase 1, cytoplasmic Thioredoxin reductase 2, and mitochondrial Ubiquilin-1 in cells treated with DOX when compared to DOX alone (Table 1). Overall, our proteomics profile confirms that MSPPOH worsened DOX-induced EndMT, inflammation, apoptosis and oxidative stress.

### CYP epoxygenase inhibitor exacerbates doxorubicin-induced endothelial toxicity in vivo in zebrafish model

**Fig. 6:**
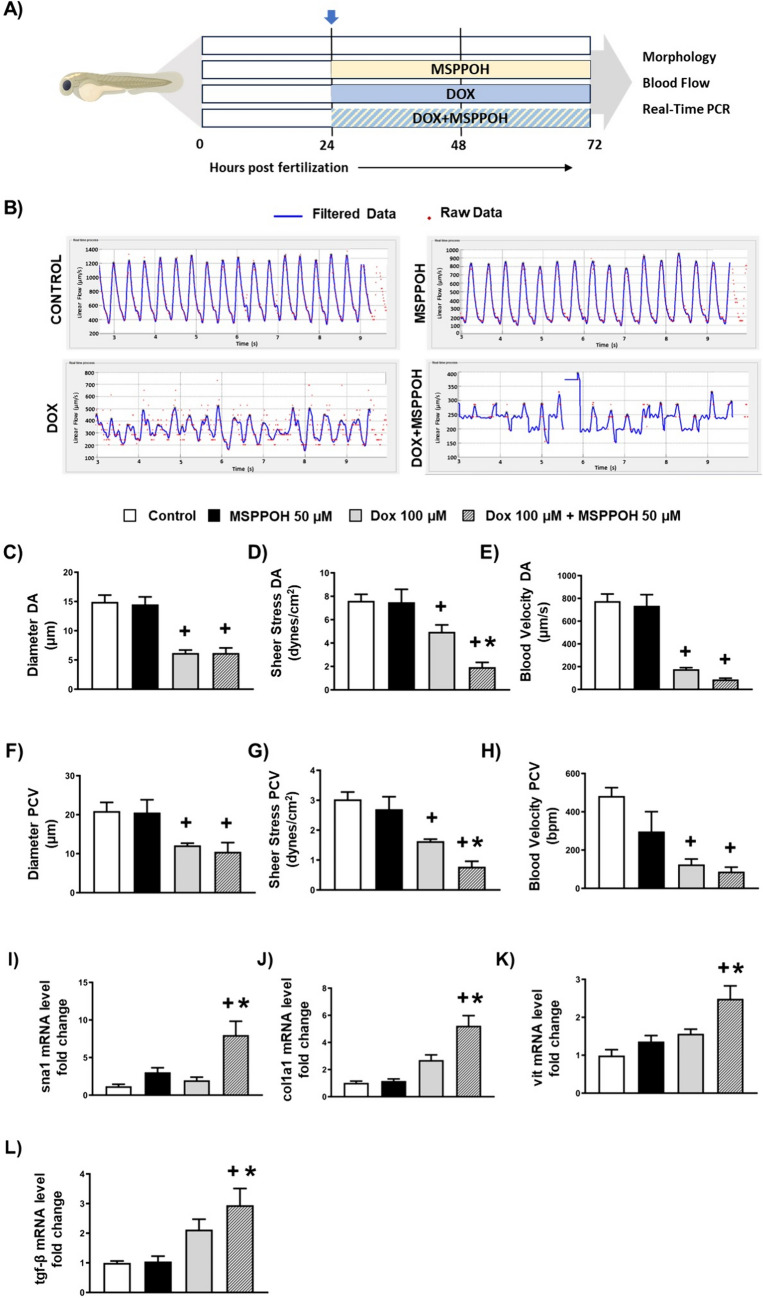
Doxorubicin-induced endothelial damage was exacerbated by CYP epoxygenase inhibitor in vivo. **A** Scheme of study design for investigating the effect of MSPPOH in DOX-induced cardiotoxicity. Microzebralab software was used to analyze blood flow. **B** Representative images of the graphs from Microzebralab depicting blood flow vs time of various treatment groups. **C** Dorsal Aorta (DA) Diameter, **D** DA Sheer stress and **E** DA blood velocity. **F** Posterior Cardinal Vein (PCV) Diameter, **G** PCV Sheer stress and **H** PCV blood velocity. **I**–**L** Quantification of mRNA expression levels by qRT-PCR. **I** snail-1 (sna-1), **J** collagen1a1 (col1a1), **K** vimentin (vit) and **L** transforming growth factor-β (tgf-β). Results are shown as means ± SEM. One-way analysis of variance (ANOVA) followed by Tukey Kramer’s post hoc multiple comparison test was used to assess if treatment with MSPPOH displayed a significant difference from the control or DOX-treated group. ^+^*p* < 0.05 vs control; *p < 0.05 vs DOX

We sought to determine whether the DOX-induced endothelial toxicity is also aggravated by CYP epoxygenase inhibition in vivo. To do this, we treated embryonic zebrafish with DOX in the presence and absence of MSPPOH at 24 hpf (Fig. 6A). We then assessed the cardiovascular function using the Viewpoints MicroZebralab at 72 hpf (Fig. 6A). Notably, we found that DOX treatment in zebrafish resulted in a significant decrease in the diameter, sheer stress, and blood flow velocity of both the Dorsal Aorta (DA) and the Posterior Cardinal Vein (PCV) when compared to the control group (Fig. 6C–H). Interestingly, while the combination of MSPPOH and DOX caused a modest reduction in the diameter and velocity of both the DA and PCV (Fig. 6C, E, F, H), it significantly worsened the shear stress of both the DA and PCV when compared to DOX treatment alone (Fig. 6D, G). To confirm if MSPPOH exacerbates DOX-induced endothelial toxicity in zebrafish at the molecular level, we conducted qRT-PCR tests to measure the expression of EndMT markers. Consistent with our in vitro data, we found that a combination of MSPPOH and DOX led to a significant upregulation of EndMT markers, including sna1, col1a1, vimentin and tgfβ when compared to DOX alone and control (Fig. 6I, J, K, L). Together, our data indicate that MSPPOH aggravates DOX-induced endothelial damage in zebrafish.

### Doxorubicin-induced cardiac injury is worsened by CYP epoxygenase inhibitor in vivo in zebrafish

**Fig. 7:**
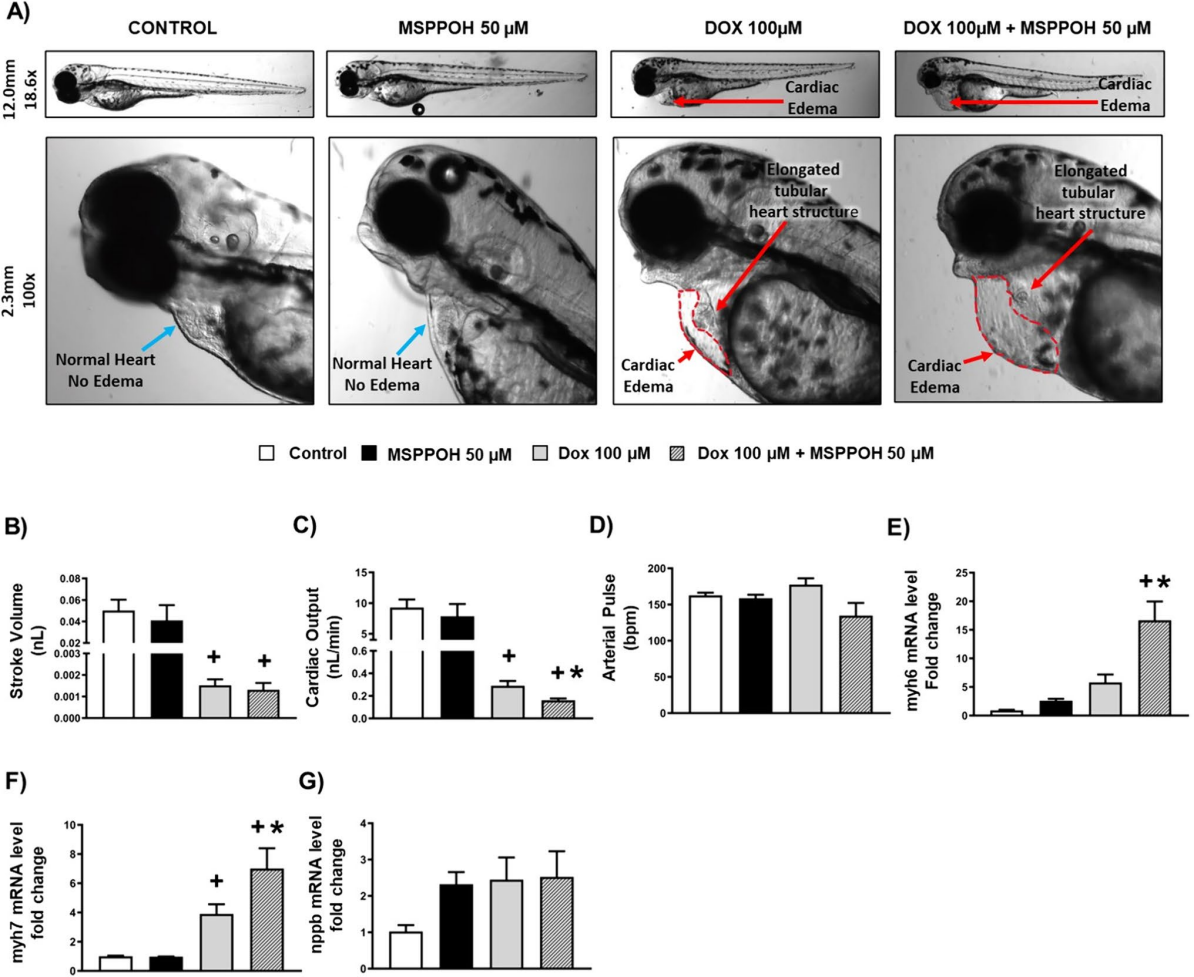
DOX-induced cardiac injury is worsened by CYP epoxygenase inhibitor in zebrafish. **A** Representative images of zebrafish **B** Stroke volume (nL), **C** Cardiac Output (nL/min), **D** Arterial Pulse (bpm). **E**–**G** Quantification of mRNA expression levels by qRT-PCR. **E** myh6, **F** myh7 and **G** nppb. Results are shown as means ± SEM. One-way analysis of variance (ANOVA) followed by Tukey Kramer’s post hoc multiple comparison test was used to assess if treatment with MSPPOH displayed a significant difference from the control or DOX-treated group. ^+^p < 0.05 vs control; *p < 0.05 vs DOX

**Fig. 8:**
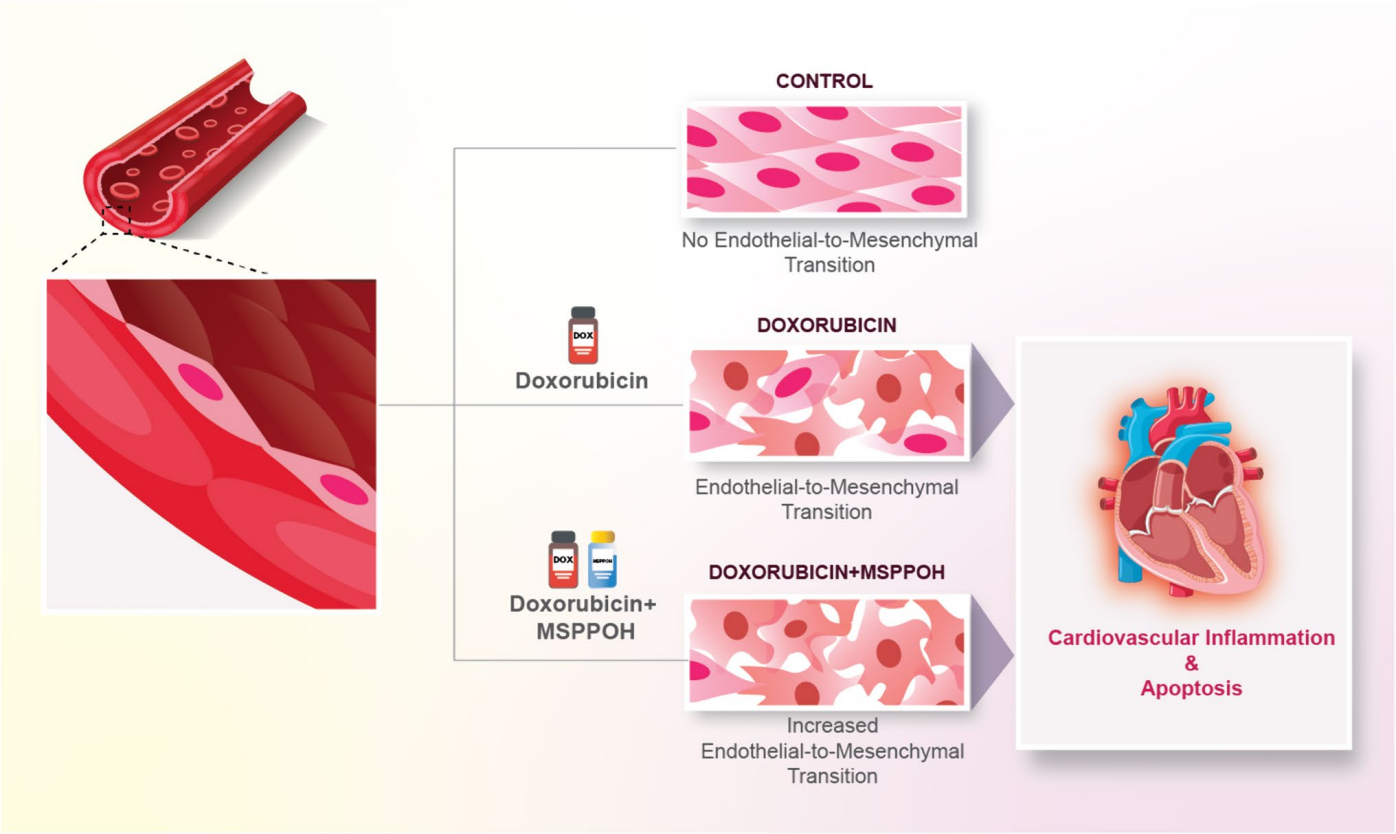
Schematic of the detrimental effects of DOX and MSPPOH on the endothelial cells and cardiovascular function

Given (i) the detrimental role of endothelial toxicity and EndMT in cardiac function [7], and (ii) the inhibition of CYP epoxygenases worsened DOX-induced endothelial toxicity and EndMT, we aimed to explore whether CYP epoxygenase inhibitor, MSPPOH, also exacerbated cardiac injury induced by DOX in vivo in our embryonic zebrafish model. To test this, we examined the cardiac morphology, cardiac function parameters, and cardiac injury markers in our embryonic zebrafish treated with DOX with or without MSPPOH. Importantly, we found that DOX treatment caused cardiac edema, reduced stroke volume, and cardiac output, as well as it upregulated the expression of myh6 and myh7 in our embryonic zebrafish model compared to the control group (Fig. 7A–C, E, F and Supplementary Table 3). Interestingly, the incidence and size of cardiac edema were further exacerbated in our zebrafish treated with a combination of MSPPOH and DOX compared to DOX alone (Fig. 7A and Supplementary Table 3). Additionally, MSPPOH significantly reduced cardiac output (Fig. 7C) and upregulated the expression of cardiac injury markers, myh6 and myh7 (Fig. 7E, F), in zebrafish treated with DOX compared to DOX alone. However, neither DOX nor MSPPOH significantly changed arterial pulse and nppb expression in our embryonic zebrafish model (Fig. 7D, G). Overall, our data suggests that MSPPOH exacerbates DOX-induced cardiovascular toxicity in our embryonic zebrafish model (Fig. 8).

## Discussion

DOX is a potent anticancer agent used to combat a wide range of malignancies [15]. However, the detrimental cardiotoxic effect of DOX limits its clinical potential and impairs the quality and duration of the post-chemotherapy life of cancer patients [8]. While previous studies primarily focused on the direct impact of DOX on cardiomyocytes, recent studies suggest that DOX toxicity may start earlier and affect the endothelium [1, 14, 23, 31]. However, the molecular mechanism responsible for endothelial toxicity is still unclear.

In the present study, we show that DOX promotes a phenotypic transition of human endothelial cells into mesenchymal traits in a process known as EndMT. We also provide evidence suggesting that DOX-induced EndMT was associated with the upregulation of pro-inflammatory, oxidative stress, and apoptotic markers in our human endothelial cells. Our work agrees with a previous report demonstrating that EndMT promotes the upregulation of pro-inflammatory, oxidative stress, and apoptotic markers to contribute to DOX-induced endothelial toxicity [14]. On the other hand, it has been shown that reducing EndMT improves endothelial function and reduces endothelial and cardiac inflammation, oxidative stress, and apoptosis in a model of DOX-induced cardiotoxicity [31]. Thus, it is likely that EndMT plays a critical role in DOX-induced endothelial dysfunction and cardiotoxicity.

The present study also sheds light on the vital role of CYP epoxygenase in the maintenance of endothelial health. We show that inhibiting CYP epoxygenases using a selective CYP epoxygenase inhibitor, MSPPOH, further exacerbates DOX-induced EndMT, inflammation, oxidative stress, and apoptosis in our human endothelial cells. This finding is in agreement with a recent report showing that loss of endothelial CYP epoxygenase function induces endothelial inflammation, oxidative stress, and apoptosis in mouse endothelial and aortic cells [28]. Based on these findings, it is reasonable to assume that CYP epoxygenase plays a physiologic protective effect on DOX-induced endothelial toxicity. Consistent with this, a previous study has demonstrated that the gain of endothelial CYP epoxygenase function, like overexpression of CYP2J, reduces endothelial inflammation, oxidative stress, and apoptosis in a mouse model of cardiovascular injury [2]. Thus, our data suggest that suppressing endothelial CYP epoxygenase function promotes DOX-induced endothelial toxicity and could contribute to DOX-induced cardiovascular toxicity.

Growing evidence indicates that EndMT and subsequent endothelial toxicity are known to disrupt the endothelial barrier, increase vascular permeability, and lead to cardiac edema and inflammation [14, 19, 47]. Thus, it is likely that suppression of CYP epoxygenase could also exacerbate DOX-induced cardiovascular toxicity. Consistent with this notion, we found that suppression of CYP epoxygenase exacerbates endothelial toxicity, lowers shear stress, reduces vascular function, increases cardiac edema, and lowers cardiac output in our zebrafish model of DOX-induced cardiotoxicity. This finding is congruent with a recent observation showing that the downregulation of endothelial CYP2j induces endothelial inflammation and toxicity and leads to cardiac injury [45]. On the other hand, overexpression of endothelial Cyp2j reduces endothelial toxicity, attenuates vascular dysfunction, decreases cardiac inflammation, and improves myocardial function in numerous models of cardiac injury [46, 48].

An important limitation of our study is that we used EA.hy926 cells as a model for endothelial cells. While EA.hy926 cell line is a somatic hybrid of primary human umbilical vascular endothelial cells and A549 cancer cells, EA.hy926 cell line exhibits a specific characteristic of vascular endothelial cells structure like the presence of Weibel-Palade bodies and function, such as, the regulation of inflammation, blood pressure, homeostasis and angiogenesis [4, 22]. Also, although our findings indicate that MSPPOH promotes EndMT in DOX-treated cells, we did not confirm our findings using different endothelial cell lines to rule out cell line-specific differences. Thus, our findings should be confirmed in other endothelial cell models like primary endothelial cells such as human aortic and coronary artery endothelial cells [5]. Finally, while MSPPOH worsens endothelial toxicity and exacerbates DOX-induced cardiovascular dysfunction in our zebrafish model, we do not have experimental evidence demonstrating that endothelial toxicity contributes to DOX-induced cardiotoxicity.

## Conclusion

In summary, our data indicate that increased EndMT, along with endothelial inflammation, oxidative stress, and apoptosis, play a vital role in DOX-induced endothelial dysfunction and cardiovascular toxicity. We also show that suppression of endothelial CYP epoxygenase promotes DOX-induced endothelial toxicity to contributes to DOX-induced cardiovascular toxicity. Given that: (a) there is a need for pharmaceutical targets to minimize DOX-induced cardiotoxicity while maintaining anticancer effectiveness, and (b) EndMT is linked to cancer growth, metastasis, and tumor cell resistance to DOX [6, 7], strategies that target the EndMT, such as activating the CYP epoxygenase system, may help reduce DOX-induced cardiotoxicity while preserving and/or improving its anticancer effectiveness. Thus, further research is warranted to test the protein expression and the activity of enzymes that regulate EETs in endothelial cells as well as to explore whether CYP epoxygenase pathway may prove to be a potential target to alleviate cardiotoxicity in patients undergoing chemotherapy.

## Supplementary Information

Below is the link to the electronic supplementary material.

Supplementary file1 (DOCX 22 KB)

Supplementary file2 (DOCX 20 KB)

Supplementary file3 (DOCX 14 KB)

## Data Availability

No datasets were generated or analysed during the current study.
