# Supplementary material for: Inhibition of cytochrome P450 epoxygenase promotes endothelium-to-mesenchymal transition and exacerbates doxorubicin-induced cardiovascular toxicity
Source: Mol Biol Rep. 2024 Jul 27;51(1):859. doi: 10.1007/s11033-024-09803-z (PMC11283412; doi:10.1007/s11033-024-09803-z)
Supplement: Supplementary file 1 — Supplementary file1 (DOCX 22 KB) [file 11033_2024_9803_MOESM1_ESM.docx]

| **Cardiac Parameter** | **Formula** |
| --- | --- |
| Mean Arterial Blood Velocity | v^**^ |
| Vessel Diameter | D^**^ |
| Vessel Radius | $r= \frac{D}{2}$ |
| Cardiac Output | Π r^2^ v * time |
| Stroke Volume | CO/HR |
| Shear stress | *​*$\text{τ=}\frac{\text{4}\text{⋅}\text{μ}\text{⋅}\text{V}\text{​ }}{D}$, where *μ* = 4 × 10^−3^ Pas |

^**^Analyzed from high-frame-rate (HFR) video using Microzebralab software
