# Supplementary material for: Inhibition of cytochrome P450 epoxygenase promotes endothelium-to-mesenchymal transition and exacerbates doxorubicin-induced cardiovascular toxicity
Source: Mol Biol Rep. 2024 Jul 27;51(1):859. doi: 10.1007/s11033-024-09803-z (PMC11283412; doi:10.1007/s11033-024-09803-z)
Supplement: Supplementary file 2 — Supplementary file2 (DOCX 20 KB) [file 11033_2024_9803_MOESM2_ESM.docx]

# PRIMER SEQUENCES

| 1 | h-b-Actin | CTG GCA CCC AGC ACA ATG | GCC GAT CCA CAC GGA GTA CT |
| --- | --- | --- | --- |
| 2 | h-SNAIL | CCTCGACCACTATGCCGCGC | GGATGGCTGCCAGCAGGTGG |
| 3 | h-NLRP3 | CTTCTCTGATGAGGCCCAAG | GCAGCAAACTGGAAAGGAAG |
| 4 | h-IL-18 | GCGTCACTACACTCAGCTAA | GCCTAGAGGTATGGCTGTAA |
| 5 | h-TWIST1 | GACAAGCTGAGCAAGATTCAGA | TGAGCCACATAGCTGCAG |
| 6 | h-SLUG | CATGCCTGTCATACCACAAC | GGTGTCAGATGGAGGAGGG |
| 7 | h-N-CADHERIN | CTCCTATGAGTGGAACAGGAACG | TTGGATCAATGTCATAATCAAGTGCTGTA |
| 8 | h-VCAM | CCAGTTGAAGGATGCGGGAG | AGCACGAGAAGCTCAGGAG |
| 9 | h-IL-1B | CCA GGG ACA GGA TAT GGA GCA | TTC AAC ACG CAG GAC AGG TAC AG |
| 10 | h-IL-6 | GGT ACA TCC TCG ACG GCA TCT | GTG CCT CTT TGC TGC TTT CAC |
| 11 | h-CD31 | AACCCACTCCCCGACCTAGA | CCAGACACCATTCCAAAACC |
| 12 | h-Α-SMA | ACTGAGCGTGGCTATTCCTCCGTT | GCAGTGGCCATCTCATTTTCA |
| 13 | h-COL1A1 | GGCAACAGCCGCTTCACCTAC | GCGGGAGGACTTGGTGGTTTT |
| 14 | h-TGF-Β | TACCTGAACCCGTGTTGCTCT | ATCGCCAGGAATTGTTGCTG |
| 15 | h-SMA22 | AGAATGATGGGCACTACCGTG | CTGTTGCTGCCCATCTGAAG |
| 16 | h-VCAM | GGAAAAAGGAATCCAGGTGGAGA | ACACTTGACTGTGATCGGCTTCC |
| 17 | h-ICAM | CTCCAATGTGCCAGGCTTG | CAGTGGGAAAGTGCCATCCT |
| 18 | h-Vimentin | CAGGCAAAGCAGGAGTCCA | AAGTTCTCTTCCATTTCACGCA |
| 19 | h-DR4 | GTGCTGTCCCATGGAGGTA | AGTACATCTAGGTGCGTTCCTG |
| 20 | h-BAX | CCCTTTTGCTTCAGGGTTTC | TCTTCTTCCAGATGGTGAGTG |
| 21 | h-BCL-XL | GGCGGCTGGGATACTTTTGT | CTCGGCTGCTGCATTGTTC |
| 22 | h-CXCR2 | TGCATCAGTGTGGACCGTTA | CCGCCAGTTTGCTGTATTG |
| 23 | h-TRAIL-1 | GATCGATGTGGTCAGAGCTGG | TGTGGATCGAGGCGTTCC |
| 24 | h-CYP2B6 | TTAGGGAAGCGGATTTGTCTT | GGAGGATGGTGGTGAAGAAGA |
| 25 | h-CYP2C8 | CACCCAGAGGTCACAGCTAAAGT | CATGTGGCTATCCTGCAT |
| 26 | h-CYP2C9 | GCCTGAAACCCATAGTGGTG | GGGGCTCAAAATCTTGATG |
| 27 | h-CYP2C19 | TGCTCTCCTTCTCCTGCTGAA | TGCCAACGACACGTTCAATC |
| 28 | h-CYP2J2 | GAAATGAGGGTCAAAAGGCTGT | GAGCTTAGAGGAACGCATTCAG |
| 29 | z-rpl13a | TCTGGAGGACTGTAAGAGGTATGC | AGACGCACAATCTTGAGAGCAG |
| 30 | z-sna1 | ACCTGCTCTCGCACCTTTAGT | TGATGCGTCATCCTTCTCCTG |
| 31 | z-vimentin | GGAAAAGAGCAAAGTGGAGGT | GATCTGCATCTCAGCAAGTTC |
| 32 | z-tgfb1b | GGGTTGCTGTGTTAGAAGTC | CAACTGTTCCACCTTATGCTG |
| 33 | z-col1a1a | GTACTGGATTGACCCTGACC | CATACTCGAACTGGAAGCCA |
| 34 | z-myh-7 | TGTATTTAGGAGGCTCTGGGTG | ACTGTCTGCTTTGCTGTTGGTC |
| 35 | z-myh-6 | TGAAGACCTGAGAAGGCAAC | CAGTTCCTCGGTTCTCTGAA |
| 36 | z-Nppb | CATGGGTGTTTTAAAGTTTCTCC | CTTCAATATTTGCCGCCTTTAC |
