# Supplementary material for: Inhibition of cytochrome P450 epoxygenase promotes endothelium-to-mesenchymal transition and exacerbates doxorubicin-induced cardiovascular toxicity
Source: Mol Biol Rep. 2024 Jul 27;51(1):859. doi: 10.1007/s11033-024-09803-z (PMC11283412; doi:10.1007/s11033-024-09803-z)
Supplement: Supplementary file 3 — Supplementary file3 (DOCX 14 KB) [file 11033_2024_9803_MOESM3_ESM.docx]

**Supplementary Table 3: Survival, Hatching and Edema rate in Zebrafish treated with Doxorubicin and MSPPOH**

|  | **Survival rate (%)** |  | **Hatching Rate (%)** |  | **Edema (%)** |  |
| --- | --- | --- | --- | --- | --- | --- |
| **CONTROL** | 100%±0 |  | 100%±0 |  | 0%±0 |  |
| **DOX** | 96.67%±1.05 |  | 90%±1.83 |  | 60%±3.65 |  |
| **DOX+MS** | 96.67%±1.05 |  | 96.67%±1.05 |  | 96.67%±1.05 |  |
| **MS** | 100%±0 |  | 100%±0 |  | 0%±0 |  |
